# Hypercalcemia of Malignancy Complicated by Osteonecrosis of the Jaw Treated With Cinacalcet

**DOI:** 10.1210/jcemcr/luad105

**Published:** 2023-09-02

**Authors:** Libia Vasquez, Tiffany Cortes

**Affiliations:** Department of Medicine, Division of Endocrinology, University of Texas Health Science Center at San Antonio, San Antonio, TX 78229; Department of Medicine, Division of Endocrinology, University of Texas Health Science Center at San Antonio, San Antonio, TX 78229; Sam and Ann Barshop Institute for Longevity and Aging Studies, University of Texas Health Science Center at San Antonio, San Antonio, TX 78229, USA; Geriatric Research, Education, and Clinical Center, South Texas Veterans Health Care System, San Antonio, TX 78229, USA

**Keywords:** hypercalcemia, malignancy, therapy

## Abstract

Hypercalcemia of malignancy (HCM) is a common complication seen in patients with cancer and is associated with high morbidity and mortality. Current long-term medical therapy for HCM focuses on inhibiting bone resorption with bisphosphonates or denosumab, which have the rare complication of osteonecrosis of the jaw. This case illustrates cinacalcet as an effective therapy for severe HCM resulting from PTH-related peptide in the setting of osteonecrosis of the jaw. Although the mechanism of action remains unclear, cinacalcet has been successful in other HCM cases even if not associated with elevated PTH-related peptide.

## Introduction

Endocrinologists are frequently consulted for evaluation and management for hypercalcemia of malignancy (HCM). The mechanisms by which HCM occur are elevated PTH-related protein (PTHrP), cytokine-induced local osteolysis, tumoral production of 1,25-dihydroxyvitamin D, or ectopic PTH. Initial workup for HCM involves checking a PTH level to rule out primary hyperparathyroidism or ectopic PTH production. Subsequent evaluation with PTHrP, 25-hydroxyvitamin D, and 1-25-dihydroxyvitamin D levels aid in determining HCM from non-PTH etiologies.

Ideal management of HCM is treatment of the underlying malignancy until remission is achieved. Because the treatment course may be prolonged or remission is not achieved, medications can be required to control calcium. Immediate hypercalcemia treatment is focused on promoting renal calcium excretion with IV fluids and the possible addition of loop diuretics or calcitonin [1]. Continued therapy for non-PTH-mediated HCM include antiresorptive medications, bisphosphonates, and denosumab, which carry the risk of osteonecrosis of the jaw (ONJ). Antiresorptive medications in treatment of HCM are given at higher and more frequent dosages compared with treatment regimens for osteoporosis. This case presents a patient with severe hypercalcemia treated with antiresorptive therapy, who developed ONJ complicated by osteomyelitis, and use of off-label cinacalcet treatment normalized calcium levels while imaging completed by her oncologist revealed the metastatic disease pattern had not progressed.

## Case Presentation

A 57-year-old woman with heart failure with a reduced ejection fraction (HFrEF) of 30% to 35% treated with furosemide and recurrent left-sided breast cancer, estrogen receptor-positive and human epidermal growth factor receptor 2 negative, stage 4, presented to the hospital from oncology infusion clinic because of hypercalcemia.

Her breast cancer was initially treated with bilateral mastectomy, chemotherapy (doxorubicin, cyclophosphamide, and paclitaxel), and radiation. She was then on tamoxifen, which was later changed to anastrazole, for continued management. She became uninsured after 3 years and was unable to continue follow up or medications. Eight years later, in 2017, she presented to the hospital with abdominal pain and distension resulting from tumor burden from breast cancer recurrence. Bone biopsy of a hip lesion confirmed metastatic disease. She was diagnosed with stage 4 breast cancer with bone metastasis and started on chemotherapy (exemestane, carboplatin, and paclitaxel) and denosumab 120 mg monthly (Table 1). Of note, no hypercalcemia was documented when denosumab was initiated. She was referred for a dental consultation, but referral was not completed before denosumab administration. She started to have symptomatic dental issues that required extensive dental extractions in February 2020; consequently, denosumab was withdrawn.

**Table 1.** Treatment course before case presentation

Date	CCa (8.2-10.3 mg/dL)	PTH (19-88 pg/mL)	PTHrP (0-3.4 pmol/L)	Vit D-25 (30-80 ng/mL)	Treatment course
7/2018	9.3				Monthly denosumab started
11/2019	9.4			24	
2/2020					Denosumab held for dental extraction
7/2020	14.8	10	7.9	40	Admitted for hypercalcemia Denosumab restarted
3/2021	9.8				
7/2021	>13 (not corrected)				Zoledronic acid 3 mg
8/2021	10.6				
9/2021	14.2				Zoledronic acid 4 mg
12/2021	10.2				Last denosumab injection
3/2022	10.2				1 L normal saline monthly
4/2022	11.3				1 L normal saline monthly
5/2022	>13 (not corrected)				Admitted for symptomatic hypercalcemia

Ca conversion mg/dL × 0.2495 = mmol/L; PTH and PTHrP conversion pg/mL × 0.1061 = pmol/L; 25-hydroxy vitamin D conversion ng/mL × 2.5 = nmol/L

Abbreviations: Alb, albumin; Ca, calcium; CCa, corrected calcium; PTHrP, PTH-related peptide; Vit D-25, 25-hydroxy vitamin D.

During her denosumab holiday, she developed symptomatic hypercalcemia, with corrected calcium, 14.8 mg/dL (3.69 mmol/L) (normal ranges, 8.2-10.3 mg/dL; 2.05-2.57 mmol/L); PTH, 10 pg/mL (1.1 pmol/L) (normal ranges, 19-88 pg/mL; 2.01-9.34 pmol/L); PTHrP, 74.4 pg/mL (7.9 pmol/L) (normal ranges, 0-32.1; 0-3.4 pmol/L); and 25-hydroxyvitamin D 40 ng/mL (99.8 nmol/L) (normal ranges, 30-80 ng/mL; 75-200 nmol/L). Monthly denosumab was restarted in July 2020 for hypercalcemia. The patient had recurrent episodes of hypercalcemia that required additional management with 2 infusions of zoledronic acid the following year. This combination antiresorptive therapy improved her hypercalcemia.

In early 2022, she had jaw pain and swelling that prompted concerns for ONJ leading to a referral to oral maxillofacial surgery (OMFS) and cessation of denosumab therapy. She received IV fluids intermittently at the infusion center when she was due for chemotherapy. By May 2022, when she presented for her infusion, she reported nausea, vomiting, mild confusion, palpitations, and altered vision described as “silver dots.” Her calcium levels were found to be greater than 13 mg/dL (3.24 mmol/L) and she was transferred to the hospital.

## Diagnostic Assessment

On presentation to the hospital, her corrected calcium was 15.5 mg/dL (3.87 mmol/L), PTH was 22.8 pg/mL (2.4 pmol/L), and 25-hydroxyvitamin D 22.0 ng/mL (54.9 nmol/L). On physical examination, she was edentulous with bone exposure measuring 2 mm with expressible pus and no sign of volume overload or other abnormality. Repeat computed tomography maxillofacial scan with IV contrast showed extensive lytic and sclerotic changes involving the mandibular body bilaterally that was compatible with osteonecrosis and possible superimposed chronic osteomyelitis. Additionally, a pathologic fracture at the junction of the right mandibular body and angle was seen. She had a significant progression of her dental disease compared with the imaging 3 months prior. PTHrP and 1,25-dihydroxyvitamin D laboratory tests were undertaken but test results returned after the patient’s discharge.

## Treatment

OMFS, along with the internal medicine team, initiated treatment for osteomyelitis. OMFS recommended no further antiresorptive therapy in the setting of severe ONJ. Calcitonin and continuous IV fluids decreased calcium levels to 13.0 mg/dL (3.24 mmol/L). Endocrinology was consulted to determine further treatment options. Extensive discussion occurred with the patient and her adult daughter regarding her treatment history, current situation, and inability to use first-line therapy for her hypercalcemia. Considering the severity of the hypercalcemia, off-label use of cinacalcet was discussed. The primary medicine team, patient, and adult daughter agreed with trial of cinacalcet 30 mg twice a day. Calcitonin was stopped after 3 days, and IV fluids were continued at decreasing rates while she was encouraged to increase her oral water intake (Table 2). Throughout hospitalization, she continued to be on metoprolol succinate 100 mg daily and furosemide 20 mg twice daily for HFrEF.

**Table 2.** Treatment course during case presentation

Date	Ca/Alb/CCa (Ca 8.2-10.3 mg/dL)	PTH (19-88 pg/mL)	PTHrP (0-3.4 pmol/L)	Vit D-1,25 (19.9-79.3 pg/mL)	Vit D-25 (30-80 ng/mL)	Treatment course
5/3/22	15.8					Admitted
5/4/22 8:00	15.1					Calcitonin 300 U twice daily IV fluids 100 mL/h
5/4/22 11:37	15/−	22.8				
5/5/22	13.0		13.7	< 5.0	22	IV fluids 100 mL/h Calcitonin 300 U twice daily
5/6/22	12.2					IV fluid 75 mL/h Calcitonin 300 U twice daily cinacalcet 30 mg twice daily
5/7/22	12.0					IV fluids 75 mL/h Cinacalcet 30 mg twice daily ->Discontinued Calcitonin
5/8/22	11.4					IV fluids 75 mL/h Cinacalcet 30 mg twice daily
5/9/22	11.4					Cinacalcet 30 mg twice daily/IV fluids stopped
5/10/22	11.4					Cinacalcet 30 mg twice daily/discharge from hospital
1/2023	9.4					Cinacalcet changed to 30 mg once a day
5/2023	9.7					Cinacalcet 30 mg once a day

Ca conversion mg/dL × 0.2495 = mmol/L; PTH and PTHrP conversion pg/mL × 0.1061 = pmol/L; 25-hydroxy vitamin D conversion ng/mL × 2.5 = nmol/L.

Abbreviations: Alb, albumin; Ca, calcium; CCa, corrected calcium; PTHrP, PTH-related peptide; Vit D-25, 25-hydroxy vitamin D.

## Outcome and Follow-up

Her corrected calcium decreased to 11.4 mg/dL (2.84 mmol/L) with IV fluids and cinacalcet. Intravenous fluid rate was not increased because of risk of volume overload HFrEF; furosemide was continued at the same dose. As her calcium levels decreased, she felt mentally clearer, nausea and vomiting subsided, and she was able to eat and drink. Three days after initiation of cinacalcet, IV fluids were stopped, and her corrected calcium level remained stable at 11.4 mg/dL (2.84 mmol/L) (Table 2). She was discharged from the hospital on cinacalcet 30 mg twice daily. At clinic follow up 1 month later, her corrected calcium was at 10.7 mg/dL (2.67 mmol/L). She resumed her chemotherapy treatments and had noted no new adverse effects she could attribute to cinacalcet. The remainder of HCM workup laboratory tests obtained during hospitalization returned and showed a PTHrP level of 129 pg/mL (13.7 pmol/L) and 1,25-dihydroxyvitamin D < 5.0 pg/mL (<13.0 pmol/L).

Her insurance did not cover cinacalcet for this off-label use, but the county health care pharmacy allowed her to pay for her prescription on a payment plan and subsequently found that a medication coupon would make it more affordable. Five months after initiation of cinacalcet 30 mg twice daily, her corrected calcium decreased further to 9.4 mg/dL (2.35 mmol/L). Oncology continued to monitor her cancer with imaging and, on its assessment, there was no progression or remission of metastatic disease. More than a year after being followed by the endocrinology clinic, her cinacalcet was decreased to 30 mg daily. But she began to miss appointments with endocrinology. Her calcium levels were reviewed from the laboratory tests she got monthly before her chemotherapy, and it remained in the normal range. She was called when she missed appointments and confirmed she continued to take the cinacalcet 30 mg daily. Although repeat PTHrP and PTH were ordered by endocrinology, these were not drawn. OMFS continued treatment of her ONJ with further debridement procedures, and she completed IV antibiotics for osteomyelitis.

## Discussion

Zoledronic acid and denosumab are frequently used in metastatic bone disease, HCM, and osteoporosis. Because of the high and frequent dosages required of denosumab and bisphosphonates, ONJ is more common in those being treated for HCM compared with osteoporosis. The risk of ONJ is mitigated with dental hygiene and oral health monitoring in those with HCM before initiation of denosumab or bisphosphonate therapy. Yet, as shown with this case, even with planned monitoring and evaluation, adverse reactions can occur and limit the available options for management of HCM.

Cinacalcet is most used in secondary hyperparathyroidism for patients on dialysis, HCM secondary to parathyroid carcinoma, and in primary hyperparathyroidism for those who are not surgical candidates [2]. Although not widely investigated or approved, cinacalcet has been successfully used in other HCM cases. In the 2022 *The Journal of Clinical Endocrinology & Metabolism* HCM guideline, cinacalcet is primarily recommended for HCM from parathyroid carcinoma with a comment that there are case reports that its use results in decreased calcium levels in those with nonparathyroid carcinomas [3]. Callaghan and Yau (2021) described successful cinacalcet treatment in the setting of HCM refractory to conventional treatments (bisphosphonates or denosumab) with confirmed PTHrP elevations in patients with renal cell carcinoma, lung carcinomas, neuroendocrine tumors, and breast cancer [4]. Cases of HCM secondary to elevated 1,25-dihydroxyvitamin D have also been effectively treated with cinacalcet [5].

Cinacalcet lowers calcium levels through G protein-coupled transmembrane cell-surface calcium-sensing receptor, primarily located on chief cells of the parathyroid gland, activation of which inhibits PTH secretion and enhances renal urinary calcium excretion [6]. Although PTH and PTHrP share physiologic and genetic homology, cinacalcet has not been shown to consistently lower PTHrP levels to account for the calcium changes [4]. Calcitonin has been noted to increase with cinacalcet therapy, which is theorized to be the mechanism by which hypercalcemia is improved. C cells of the thyroid cell-surface calcium-sensing receptor that cinacalcet can activate can lead to increased calcitonin. Colloton et al [7] compared the calcium levels of parathyroidectomized to thyroidectomized rats after cinacalcet administration and reported that those without thyroid glands did not have reduction in their calcium levels when given cinacalcet. If the thyroid gland was intact and only the parathyroid glands were removed, cinacalcet did effectively decrease calcium levels. They concluded cinacalcet's calcium-lowering effect must be in part due to increased endogenous calcitonin secretion [7]. Sheehan et al [7] hypothesized, after evaluation of urinary calcium excretion, bone turnover, and 1,25-dihydroxyvitamin D levels that cinacalcet works by decreasing calcium intestinal absorption [8]. Because calcium intestinal absorption is the main mechanism by which 1,25-dihydroxyvitamin D leads to hypercalcemia, this mechanism can explain the effect that cinacalcet has in HCM secondary to elevated 1,25-dihydroxyvitamin D levels.

Although there is not a clear mechanism of action widely identified to explain why cinacalcet has been an effective way to normalize calcium in those with nonparathyroid carcinoma HCM, multiple cases of refractory HCM have been successfully treatment with cinacalcet [5, 7‐9]. The previously theorized mechanisms likely contributed to calcium normalization in this patient with humoral, elevated PTHrP, and osteolytic cytokine, bone metastasis, mediated hypercalcemia. Because of her heart failure diagnosis, she continued to need furosemide. Loop diuretics can help decrease calcium levels through renal excretion and, as a result, cause secondary hyperparathyroidism. As noted, she continued to have minimal production of PTH, which could be because of furosemide use, parathyroid adenoma, or the cancer itself, and cinacalcet is known to be effective at managing PTH-mediated hypercalcemia. However, as her hypercalcemia worsened and improved on the same dose of furosemide, it is unlikely this medication had a significant effect on her calcium status. For this case, cinacalcet was and continues to be an effective therapy to fully normalize calcium levels even though she has not had significant tumor burden decrease based on periodic imaging and assessment by her oncologist. Attempts were made to have her PTHrP and PTH checked but they were not repeated. It is important for endocrinologists to be aware of cinacalcet as a potential treatment for HCM especially because their expertise may be requested in cases where first-line therapies can no longer be used, as in the setting of ONJ.

## Learning Points

- Importance of early dental clinical and imaging evaluation when antiresorptive medications are possibly needed for hypercalcemia of malignancy (HCM) or bone metastasis treatment.
- Cinacalcet has been an effective, off-label, therapy for HCM resulting from elevated PTH-related peptide or 1,25 dihydroxyvitamin D.
- The mechanism of action for cinacalcet in treatment of HCM is not well understood but possible reasons for its effect include increased endogenous calcitonin levels, decreased calcium absorption, and increased calcium urinary excretion.

## Contributors

All authors made individual contributions to authorship. L.V. and T.C. were involved in the diagnosis and management of this patient and in manuscript submission.

## Data Availability

Data sharing is not applicable to this article as no datasheets were generated or analyzed during the current study.

## Abbreviations

HCM: hypercalcemia of malignancy
HFrEF: heart failure with a reduced ejection fraction
OMFS: oral maxillofacial surgery
ONJ: osteonecrosis of the jaw
PTHrP: PTH-related protein
